# Using pedometers to increase physical activity in overweight and obese women: a pilot study

**DOI:** 10.1186/1471-2458-9-309

**Published:** 2009-08-25

**Authors:** Sebely Pal, Cheryl Cheng, Garry Egger, Colin Binns, Robert Donovan

**Affiliations:** 1 School of Public Health, Curtin Health Innovation Research Institute, ATN Centre for Metabolic Fitness, Curtin University of Technology, Perth, Western Australia, Australia; 2 School of Health Sciences, Deakin University, Melbourne, Australia

## Abstract

**Background:**

Most public health guidelines recommend that adults participate in 30 minutes of moderate intensity physical activity on most days of the week. Establishing new ways to achieve these targets in sedentary populations need to be explored. This research evaluated whether the daily use of pedometers could increase physical activity and improve health outcomes in sedentary overweight and obese women.

**Methods:**

Twenty six overweight and obese middle-aged women were randomized into two groups: The control group was not able to record their steps daily, whilst the pedometer group, were asked to record the number of steps on a daily basis for 12 weeks.

**Results:**

Our data showed that the pedometer group significantly increased their steps/day, by 36%, at the end of the 12 weeks, whereas the control group's physical activity levels remained unchanged. There was no significant difference in weight or body fat composition in the pedometer group compared to the control group. However, there was a significant decrease in systolic blood pressure in the pedometer group (112.8 ± 2.44 mm Hg) compared to the control group (117.3 ± 2.03 mm Hg) (p = 0.003).

**Conclusion:**

In conclusion, this pilot study shows that the combination of having step goals and immediate feedback from using a pedometer was effective in increasing physical activity levels in sedentary overweight and obese women.

**Trial registration:**

ACTRN12609000176268

## Background

Obesity has reached epidemic proportions globally, with more than 1 billion adults overweight and at least 300 million of these clinically obese [1]. Obesity carries with it significantly higher risks for developing diabetes, hypertension, stroke, coronary artery disease, sleep apnoea, joint and respiratory problems [2-4].

Studies have shown that exercise can elicit improvements in cardiovascular fitness, body composition, blood lipid profile and retention of essential muscle mass [5]. Regular physical activity also improves, mental health states [6], blood pressure [7], type 2 diabetes [8], protects against some cancers [9] and the risk of osteoporosis [10]. Despite increased knowledge about the benefits of exercise, weight loss and a healthy diet for psychological and physiological well-being, motivating sedentary overweight obese adults is a difficult task. Even those who initiate changes often find it difficult to comply with a program for any length of time, most falling back into their old inactive habits after an intervention ends [11]. Most public health guidelines recommend that adults participate in 30 minutes of moderate intensity physical activity on most, if not all days of the week [12-15]. However, the 2008 US Physical Activity Guidelines Advisory Committee recommends that overweight/obese adults should accumulate at least 60 minutes of activity on most days of the week [16]. Ensuring that a sufficient amount of physical activity has been accumulated daily to attain health benefits can be very difficult, especially in the overweight and obese. Therefore, establishing new ways to achieve these higher targets need to be explored.

It is generally recommended that lifestyle-based (or home-based) increases in physical activity, as opposed to structured exercise programs (gym, walking trainers, supervised programs) are more likely to be successful in facilitating increased physical activity and weight loss over the long-term [17,18]. Studies have shown that the daily use of pedometers is associated with significant increases in physical activity [19]. Feedback from pedometer step counts has been shown to prompt behaviour change as they raise awareness of current walking behaviours [20], can be used to motivate [21], and to self-monitor [20,21]. A meta-analysis of 26 RCTs and observational studies of pedometer use in adults reported a significant increase in the number of steps walked per day [19]. However, the authors concluded that some limitations of the included studies were: 1) the studies were all of relatively short duration and the extent to which these results are durable over the long term is unknown; 2) few studies provided detailed information about their participants or evaluated more than one of the outcomes of interest 3) many interventions included the use of two or more components (eg, pedometers, step goals, diaries, counselling). Hence studies investigating pedometer use without a step goal and/or without physical activity counselling are required, including comparisons between participants who can see their daily step counts vs pedometer use in which they are blinded to their daily step counts.

The aim of this pilot study was to investigate whether the daily recording of steps using pedometers could increase physical activity in sedentary overweight and obese women. We hypothesised that the daily use of pedometers would act as prompt to increase daily physical activity in sedentary overweight and obese women. In this study middle-aged women were targeted as > 50% of this group are overweight and therefore at high risk for developing cardiovascular disease, diabetes and hypertension [2]. Women are specifically targeted in this pilot study as they generally have a large input into their families. Thus, any lifestyle changes have the potential to flow on and affect their family members (spouses, children).

## Methods

### Participants

Thirty middle aged (35 – 55 yrs old), sedentary, overweight and obese women (body mass index {BMI} > 25 and < 35 kg/m^2^) were recruited from the local community newspapers in Perth, Australia.

Interested participants were initially screened by the research assistant using a telephone questionnaire. Those who were suitable then attended a group orientation meeting where details of the study were explained before written consent was obtained. Exclusion criteria included current chronic medical and psychological disease, major systemic illness, renal failure, pregnancy, lactating or planning to become pregnant, smoking, hypothyroidism, diabetes mellitus, pre-existing heart conditions or gastrointestinal surgery and greater than two hours of moderate intensity physical activity per week. Written informed consent from each participant was obtained prior to participating in the study. The Ethics Committee of Curtin University reviewed and approved all procedures

### Experimental Protocol

In this 12 week study, overweight and obese sedentary middle aged women were randomised into two groups: a 'pedometer' group and a 'control' group. Participants in the pedometer group were told to record their pedometer steps on a daily basis for 12 weeks; those in the control group were asked to wear a sealed pedometer for 12 weeks with weekly recording.

To collect baseline data, all thirty participants were asked to wear a sealed Yamax Digi-Walker SW-200 pedometer to record the amount of steps they accumulated over a one week period. This pedometer has an overall mean absolute error of 3% [22]. Two studies have compared 13 pedometer models and found that Yamax Digiwalker SW-200 pedometer to be the one of the most accurate and suitable for research [23,24]. The accuracy of the pedometer on each participant was checked by the means of a 20 step test at the outset, the acceptance criteria being +/-2 steps. Participants wore the pedometer clipped to their clothing at the waist, centred over the foot. Thirty participants were then randomised to two groups by the research assistant using a blocked randomisation method. The control group wore a sealed pedometer for 12 weeks and therefore had no knowledge of their record of steps/day. The pedometer was sealed with tape so that the number of steps/day was concealed. This seal was only broken once per week by the participant so that the total weekly steps could be recorded by them on a calendar provided. They were then asked to reset the pedometer to zero each week and seal the pedometer with tape. Participants signed a form to acknowledge that they understood these instructions and would abide by them. The pedometer group wore an unsealed pedometer. Hence they were able to open and observe their number of steps performed throughout the day. Participants in the pedometer group were instructed to record accumulated steps/day on a calendar and to reset the pedometer to zero each day.

At baseline, both groups were then given the National Australian Physical Activity Guidelines [12]. At baseline, information from these guidelines such as intensity, duration, prompts for exercise (ie, stairs or elevators) and relapse prevention etc were reviewed with the participants. The pedometer group was also encouraged to reach a daily step goal of 10,000 steps/day. No step goals were set for the control group. At baseline, participants from both groups were encouraged to initially set small achievable goals like 10 minute walks and then to gradually increase the goal each week to at least 30 min/day. No further advice was given to either the pedometer or control group regarding physical activity after this initial consultation. Physical activity was assessed at baseline and at 12 weeks using short-form International Physical Activity Questionnaire (IPAQ), which provides information on the time spent walking, in moderate physical activity, in vigorous physical activity and total physical activity in (MET·min/wk) (MET = Metabolic Equivalent Task) in a usual week. This version of the IPAQ has been found to be valid and reliable [25].

Participants were asked to maintain their usual dietary intake throughout the duration of the study. The maintenance of dietary intake over the course of the trial was monitored through the completion of 3-day food diaries, which included two week days and one weekend day, at the start and finish of the study. Energy and macronutrient intakes from the participants' combined food records were calculated using Food Works (Version 3 Xyris Software, 2002) based on data from the AUSNUT database.

#### Physical examination

Participants were assessed at baseline and 12 weeks. They underwent a brief physical examination, including resting heart rate (HR) and blood pressure, weight, waist and hip measurements. Height measurements were taken using a mechanical stadiometer (Surgical and Medical Products, Hills, Australia. BMI (kg/m^2^) was calculated from weight and height measurements. Waist (at umbilicus) and hip circumference were measured from which Waist to Hip ratio (WHR) was calculated. Weight measurements were taken using Tanita scales (UM-018 Digital Scales, Tanita Corporation, Tokyo, Japan), with patients dressed in light clothing without shoes. Body composition was also measured using the RJL Systems BIA – 101 Body Composition Analyzer (USA). These standardized protocols have been established in our laboratory.

### Statistical analysis

Statistical analysis was conducted using SPSS 17 for Windows (SPSS Inc., Chicago, IL). Data are expressed as mean (SEM) and assessed for normality. Comparison of baseline characteristics between each group was undertaken by one way analysis of variance. Differences within groups were determined using a two-sided paired t-test. Using one-way analysis of covariance with the baseline data as the covariate, differences between groups at week 6 and 12 were conducted. Statistical differences were analysed further by post-hoc analysis using the Least Square Differences (LSD) method. Statistical significance was considered at p < 0.05. Sample size calculation was based on a predicted change of 20% in steps/day between control and groups as suggested in previous studies [19,26,27]. Assuming a standard deviation of 20%, a sample size of 12 participants per group provides sufficient power (80%) to detect changes at the 5% significance level. A total of 30 participants were recruited to ensure adequate numbers in the event of participants choosing to withdraw from the study.

## Results

A total of 30 participants (15 participants/group) were initially recruited and started the study. However a total of twenty six completed the study. Four participants withdrew from the study (2 participants per group) due to personal issues. Two participants withdrew in week 2 from the pedometer group and the other two from the control group withdrew at week 3 and 4. Results were reported on the remaining 26 participants, with 13 participants in the pedometer group and 13 participants in the control group. Baseline characteristics of participants, such as steps/day, height, weight, age did not significantly differ between the groups (Table 1).

**Table 1:** Mean demographic data at baseline for the control and pedometer groups

***Group***	***Pedometer (n = 13)***	***Control*** ***(n = 13)***	***P value***
Age (years)	42 ± 9.2	44 ± 6.9	0.287

Weight (kg)	77.84 ± 1.91	77.16 ± 2.45	0.543

Height (cm)	161.33 ± 1.36	164.19 ± 1.88	0.676

BMI (kg/m^2^)	29.92 ± 0.68	28.60 ± 0.75	0.423

Mean steps/day	6242 ± 541	6574 ± 606	0.430

Values are represented as mean ± s.e.m

*Denotes significance at p < 0.05

*P-values *represent the comparison of control group vs pedometer group at baseline

**Figure 1:**
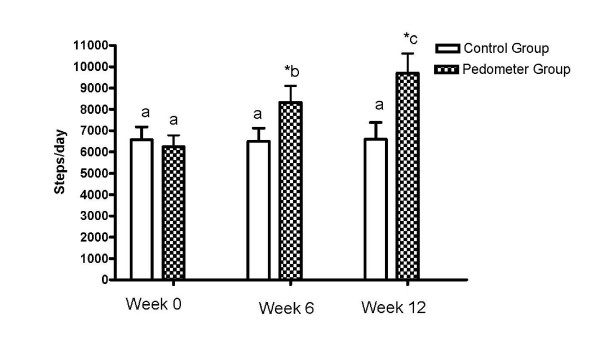
**Average number of daily steps taken at baseline, week 6 and week 12 by the control and pedometer groups**. The average steps/day were measured at baseline, week 6 and week 12 in both groups. Data is expressed as means ± SEM. Different letters above bar graphs indicate significance at p < 0.05. * Significant difference (*P *< 0.05) within group (baseline versus 6 weeks). * Significant difference (*P *< 0.05) within group (baseline versus 12 weeks).

Figure 1 shows no significant difference in the number of steps at baseline between the two groups. However, there was a significant increase in the number of steps with the pedometer group versus the control group at 6 and 12 weeks intervention (p = 0.04 and p = 0.03, respectively). At 12 weeks, the pedometer group had a 32% higher number of steps/day than the control group. The control group remained unchanged in the number of steps during the 12-week intervention. For the pedometer group, the daily average number of steps at weeks six (8321 ± 884 steps per day) and twelve (9703 ± 921 steps per day) were significantly higher than the baseline daily average of 6242 ± 541 steps per day (p = 0.046 and p = 0.035, respectively). At week twelve, the pedometer group was taking an average of 3461 steps per day more (36% increase) than at baseline.

Table 2 shows the anthropometric and blood pressure measurements at baseline and 12 weeks of the 2 groups. There was no significant differences within groups or between groups in waist, BMI, waist/hip ratio, HR or % body fat at 12 weeks. Systolic blood pressure decreased significantly by 4.7% at week 12 compared with baseline in the pedometer group (p = 0.018). The pedometer group was found to have a significantly lower systolic blood pressure (112.8 ± 2.44 mm Hg) at 12 weeks compared to the control group (117.3 ± 2.03 mm Hg) (p = 0.003). There were no significant changes to diastolic blood pressure (baseline versus 12 weeks) or between groups at 12 weeks.

**Table 2:** Anthropometric, resting heart rate and blood pressure at baseline and week 12 for the control and pedometer groups.

***Variable***	***Pedometer***		***Control***		***P-value***
		
	***Baseline***	***Week 12***	***Baseline***	***Week 12***	***Week 12***
Weight (kg)	77.84 ± 1.91	77.92 ± 2.28	77.16 ± 2.45	76.94 ± 2.55	0.799

Height (cm)	161.33 ± 1.36	161.35 ± 1.46	164.19 ± 1.88	163.78 ± 1.85	0.366

BMI (kg/m^2^)	29.92 ± 0.68	30.08 ± 0.80	28.60 ± 0.75	28.66 ± 0.81	0.962

Waist (cm)	97 ± 2.24	97.35 ± 3.16	95.5 ± 2.98	95.4 ± 2.72	0.820

Hip (cm)	110.62 ± 2.32	112.44 ± 2.68	109.24 ± 1.92	110.69 ± 1.75	0.768

WHR	0.88 ± 0.01	0.86 ± 0.01	0.87 ± 0.02	0.86 ± 0.02	0.637

% Body Fat	41.73 ± 0.79	41.6 ± 0.97	41.01 ± 1.11	40.46 ± 1.13	0.573

Resting Heart Rate	60.1 ± 2.34	62.3 ± 2.27	61.8 ± 1.93	62.5 ± 2.56	0.430

BP Systolic (mm Hg)	118.3 ± 2.55	112.8 ± 2.44* ^a^	116.9 ± 2.82	117.3 ± 2.03^b^	0.003

BP Diastolic (mm Hg)	79.13 ± 2.12	75.59 ± 2.41	76.05 ± 2.39	76.97 ± 2.28	0.179

Values are represented as mean ± s.e.m

*P-values *represent the between group comparison of the control vs pedometer group at week 12.

There were no significant differences at baseline between groups.

*Significant difference (*P *< 0.05) within group (Baseline versus 12 weeks)

Significant difference (*P *< 0.05) between control group and pedometer group at 12 weeks is indicated by different letters.

Percentage body fat was measured using the RJL Systems BIA – 101 Body Composition Analyzer

Table 3 shows that there were no significant differences between the groups in the macronutrient content of their diets at baseline or at 12 weeks. Table 4 shows that time doing moderate or vigorous physical activity was not significantly different between the control and pedometer groups. However, there was a significant increase in time spent walking in the pedometer group compared to the control group at 12 weeks and from baseline. Although time spent doing moderate activity and total physical activity increased in the pedometer group versus the control group at 12 weeks this was not significant.

**Table 3:** The macronutrient composition of diets from participants 3 day food records at baseline and 12 weeks for the control and pedometer groups

***Variable***	***Pedometer *(n = 13)**		***Control *(n = 13)**		***P-value***
		
	***Baseline***	***Week 12***	***Baseline***	***Week 12***	***Week 12***
Energy (kJ)	9024 ± 1080	8854 ± 687	9497 ± 1571	9146 ± 988	0.481

Protein (g)	99.9 ± 12.5	95 ± 9.87	105.8 ± 23	97.3 ± 9.8	0.820

Fat (g)	90.5 ± 11.33	87 ± 9.7	92.9 ± 18.7	89 ± 8.8	0.571

Carbohydrate (g)	219.7 ± 30.1	213 ± 22.3	229.7 ± 32.5	219 ± 22.4	0.128

Fibre (g)	21.7 ± 1.9	23.9 ± 2.3	24 ± 2.1	24.9 ± 3.6	0.136

Values are represented as mean ± s.e.m

*P-values *represent the between group comparison of the control vs pedometer group at week 12.

There were no significant differences at baseline between groups.

There were no significant differences between groups at 12 weeks.

There were no significant within group differences.

## Discussion

In this study we tested whether the daily use of pedometers could promote increased physical activity in sedentary overweight and obese women. Our results showed that those in the pedometer group who were able to record their daily steps had a significant increase in their number of steps/day after 6 and 12 weeks compared to those in the control group who could not monitor their steps on a daily basis. Setting a step goal and the immediate feedback from the pedometer may be a key motivational factor for increasing physical activity in the pedometer group. The increase in the daily number of steps/day was mainly through walking activity as suggested by the IPAQ results (Table 4). There were no significant differences in anthropometric measurements between the pedometer and control groups.

**Table 4:** Physical activity data from the International Physical Activity Questionnaire (IPAQ) at baseline and 12 weeks for the control and pedometer groups

***Variable***	***Pedometer***		***Control***		***P-value***
		
	***Baseline***	***Week 12***	***Baseline***	***Week 12***	***Week 12***
Walking (MET·min/wk)	244.5 ± 122.2	589.3 ± 167.2* ^a^	224.6 ± 102	201.4 ± 114.6^b^	0.036

Moderate activity (MET·min/wk)	161.5 ± 44.5	214.3 ± 67.5	177.2 ± 45.5	174.6 ± 54.5	0.234

Vigorous activity (MET·min/wk)	57.8 ± 34	59.5 ± 26	62.8 ± 23.3	61.3 ± 138.3	0.146

Total activity (MET·min/wk)	365.8 ± 195.4	689 ± 295.5	455.8 ± 203.5	389 ± 175.5	0.274

Values are represented as mean ± s.e.m

*P-values *represent the between group comparison of the control vs pedometer group at week 12.

There were no significant differences at baseline between groups.

*Significant difference (*P *< 0.05) within group (Baseline versus 12 weeks)

Significant difference (*P *< 0.05) between control group and pedometer group at 12 weeks is indicated by different letters.

Our results are consistent with previous research indicating that the daily use of pedometers is effective in increasing walking behaviour. A meta-analysis of 26 RCTs and observational studies of pedometer use in adults reported a significant increase in the number of steps walked per day [19]. Participants in the RCTs who used pedometers, recorded their steps and had a step goal increased their physical activity by an average of 2491 steps per day more than pedometer users who did not have a step goal. In the meta-analysis study, pedometer users increased their number of steps by 27% over baseline [19]. Bravata et al., [19], found that step-count goal was an important predicator of increasing physical activity. Similarly in our study, those in the control group who were not given a daily step goal and unable to open their pedometers on a daily basis, had no significant increase in their physical activity over baseline. On the other hand, the pedometer group had a step goal, were able to open view their pedometer on a daily basis and record steps. Since pedometers provide a quantifiable walking measure, they enable participants to track and record progress from a baseline reference point. Studies have shown that immediate feedback from the pedometers enable participants to set realistic goals, act as an environmental cue, raise awareness of current walking behaviours [20], to motivate [21], to self-monitor [20,21], and to increase walking behaviours [7,28].

We did not observe any changes in anthropometric measures in this study, which is consistent with other studies [29]. It has been suggested that 60 to 90 min of moderate intensity exercise on most days of the week would be required for weight loss [30]. Therefore, the extra 3461 steps steps/day (equivalent to 30 min of physical activity) performed by the pedometer group may not have been adequate to see changes in anthropometric measures in this group [31]. Anthropometric measures may have remained stable, despite increased physical activity, due to nutritional compensation. However, we did not observe any increase in dietary intake at 12 weeks (Table 3). Lack of change in nutritional intake may be due to under reporting by overweight and obese groups as shown in previous studies [32,33].

Current public health guidelines suggest that ≈ 30 min of moderate-intensity activity/day on most days of the week provides health benefits and reduces risk for a range of conditions in all population groups [12-14]. However, new recommendations by the US Physical Activity Guidelines Advisory Committee suggests that overweight/obese adults should accumulate at least 60 minutes of activity on most days of the week for weight loss [16]. Brisk walking for 60 minutes is equivalent to approximately 6,000 steps [34]. This means that overweight individuals would need to accumulate an extra 6000 steps for weight loss. Therefore, new ways are required to help achieve this higher target. In our study, sedentary overweight and obese women were asked to comply with the National Australian guidelines for adults and increase their physical activity by 30 min/day. Those in the pedometer group with 6242 ± 541 steps/day at baseline, reached an average of 9703 ± 921 steps/day after 12 weeks, an increase of more than 3000 steps/day. However, overweight adults in our study would need to accumulate at least 12,000 steps per day to comply with these new guidelines and achieve weight loss. As suggested by the the US Physical Activity Guidelines Advisory Committee, energy intake (diet) must be also be considered for weight control by this particular group [16].

The increase in daily steps in the pedometer group was mainly through walking more (Table 4). It has been previously demonstrated that walking is the single easiest way for people to increase their physical activity and sustain it over the long term [35]. Walking, which is "self paced" is the most frequently reported physical activity and most preferred in the overweight and obese as it is low-impact with little risk of injury [12,36,37].

This study showed improvements in systolic blood pressure in the pedometer group after 12 weeks compared to the control group. As dietary intake in the pedometer group at 12 weeks did not significantly change from baseline we can comment that improvements in blood pressure are most likely due to an increase in physical activity in this group and unlikely to be influenced by any changes in dietary intake. In a meta-analysis of 26 RCTs and observational studies, intervention participants, who had increased their physical activity levels, had a statistically significant decrease in systolic blood pressure of 3.8 mm Hg and diastolic blood pressure of 0.3 mm Hg [19]. This reduction in systolic blood pressure was independent of decreases in BMI. Our results are also consistent with a meta-analysis of 54 studies conducted by Whelton et al. [38] showing that aerobic exercise reduced blood pressure in both hypertensive and normotensive adults. All frequencies, intensities, and types of aerobic exercise lowered blood pressure. By highlighting the health benefits of physical activity, health professionals can use such evidence to encourage overweight and obese patients, who are frustrated by an inability to lose weight, to engage in physical activity.

In this study we asked our overweight and obese participants to comply with the Australian Physical activity guidelines which advises most adults participate in 30 minutes of moderate intensity physical activity on most days. Further work is required to demonstrate whether using pedometers on a daily basis could help the overweight and obese accumulate at least 60 minutes of activity on most days of the week, thereby meeting the new recommendations for physical activity advised for this group [16]. It is possible that changes in anthropometric measures could be observed after 12 weeks in this group with 60 min targets. One of the limitations of this study was using a pedometer without a memory chip, where only a total step count for the week was available for the control group. There is evidence to suggest that despite participants not being able to see their daily step count when wearing sealed pedometers, a certain amount of reactivity (i.e. steps are higher) occurs during the first 2–3 days of monitoring under these conditions [39]. For future studies using pedometers with a memory chip which is capable of storing daily step counts over a period of seven days could be used. A study in overweight and obese adults has shown that a piezo-electric pedometer is more accurate than a Yamax Digi-Walker in those with higher BMI [40]. This type of pedometer could be used in future studies for studying walking behaviours in overweight and obese individuals. Another limitation of this study was the lack of detail in how the participants chose to perform their physical activity. It is also suggested that in addition to daily activity, exercise should be performed in bouts of 10 mins and resistance exercise should be performed two times per week [15,29]. Although those in the pedometer group reported that they had increased their walking, the duration of each walking bout is unclear. Recommendations regarding resistance training were not highlighted to the participants.

## Conclusion

In conclusion, this pilot study shows that the combination of having step goals and immediate feedback from using a pedometer was effective in increasing physical activity levels in sedentary overweight and obese women.

## Competing interests

The authors declare that they have no competing interests.

## Authors' contributions

The authors' responsibilities were as follows – CC coordinated the trial, data collection and input into the manuscript. SP conceived and designed the study, wrote the manuscript, supervised the study and the statistical analysis. GE, CB and RD had input into the writing of the grant and manuscript. All authors have read and approved the final manuscript.

## Pre-publication history

The pre-publication history for this paper can be accessed here:


